# Positive predictive value and effectiveness of measles case-based surveillance in Uganda, 2012-2015

**DOI:** 10.1371/journal.pone.0184549

**Published:** 2017-09-08

**Authors:** Fred Nsubuga, Immaculate Ampaire, Simon Kasasa, Henry Luzze, Annet Kisakye

**Affiliations:** 1 Uganda National Expanded Program on Immunization, Ministry of Health, Kampala, Uganda; 2 Makerere University School of Public Health, Kampala, Uganda; 3 World Health Organization Country Office, Kampala, Uganda; University of Queensland, AUSTRALIA

## Abstract

**Introduction:**

Disease surveillance is a critical component in the control and elimination of vaccine preventable diseases. The Uganda National Expanded Program on Immunization strives to have a sensitive surveillance system within the Integrated Disease Surveillance and Response (IDSR) framework. We analyzed measles surveillance data to determine the effectiveness of the measles case-based surveillance system and estimate its positive predictive value in order to inform policy and practice.

**Methods:**

An IDSR alert was defined as ≥1 suspected measles case reported by a district in a week, through the electronic Health Management Information System. We defined an alert in the measles case-based surveillance system (CBS) as ≥1 suspected measles case with a blood sample collected for confirmation during the corresponding week in a particular district. Effectiveness of CBS was defined as having ≥80% of IDSR alerts with a blood sample collected for laboratory confirmation. Positive predictive value was defined as the proportion of measles case-patients who also had a positive measles serological result (IgM +). We reviewed case-based surveillance data with laboratory confirmation and measles surveillance data from the electronic Health Management Information System from 2012–2015.

**Results:**

A total of 6,974 suspected measles case-persons were investigated by the measles case-based surveillance between 2012 and 2015. Of these, 943 (14%) were measles specific IgM positive. The median age of measles case-persons between 2013 and 2015 was 4.0 years. Between 2013 and 2015, 72% of the IDSR alerts reported in the electronic Health Management Information System, had blood samples collected for laboratory confirmation. This was however less than the WHO recommended standard of ≥80%. The PPV of CBS between 2013 and 2015 was 8.6%.

**Conclusion:**

In conclusion, the effectiveness of measles case-based surveillance was sub-optimal, while the PPV showed that true measles cases have significantly reduced in Uganda. We recommended strengthening of case-based surveillance to ensure that all suspected measles cases have blood samples collected for laboratory confirmation to improve detection and ensure elimination by 2020.

## Introduction

Having an effective measles surveillance system is vital in planning, prompt outbreak response, monitoring and evaluation of control measures [1]. The Uganda National Expanded Program on Immunization (UNEPI) strives to have a sensitive disease surveillance system within the Integrated Disease Surveillance and Response (IDSR) framework [2]. This system provides routine reports of suspected measles cases as part of the general system of aggregate summary reporting of notifiable diseases and other health events. This surveillance system is entirely under the control of Ministry of Health of Uganda, which is responsible for utilization of health-related information in the health sector. Uganda adopted the IDSR strategy in 2000 with the aim of creating a coordinated approach to collection, analysis, interpretation, use and dissemination of public health data to guide evidence based decision making [3]. The IDSR strategy is a combination of active and passive systems using a single infrastructure that gathers information about multiple diseases of interest [4]. The passive system of measles surveillance uses the electronic Health Management Information System (eHMIS), where routine health data are collected from all the health facilities in the country and reported to the national Ministry of Health. The active case-based surveillance system for measles with laboratory confirmation (CBS), is based in the Uganda Virus Research Institute (UVRI) and is critical in documenting measles elimination.

During passive surveillance, data are collected from suspected measles patients during visits to health centers and then reported routinely using weekly, monthly and quarterly reports [5, 6]. During reporting health workers use standard case definitions to avoid reporting non-measles cases as measles cases. This type of surveillance however, yields limited data because most of these cases may not visit health facilities [7]. These limitations within the passive surveillance system can be overcome if surveillance officers regularly visit health facilities, traditional health care delivery points and communities. This process of ensuring that all cases are notified and reported in time is called active surveillance. Also, when a disease has been targeted for eradication/elimination as occurs with measles, health workers conduct case-based investigations.

Case-based laboratory backed surveillance was rolled out in all districts of Uganda, in October 2003 [8]. The establishment of CBS demands that a case investigation form is filled for each suspected measles patient who fulfils the case definition instead of relying on the aggregate reporting in eHMIS. In addition, a blood sample is collected at first contact or within 30 days of rash onset for serological confirmation of measles infection. Laboratory confirmation of suspected cases is based on the detection of measles specific IgM in a single blood sample taken after rash onset.

The CBS also requires that all suspected outbreaks are investigated and confirmed by collecting blood samples from the first reported 5 cases [7], while other cases are line listed and classified epidemiologically. Ideally during the elimination phase, each suspected case reported through eHMIS should also be captured by CBS as they use the same case definition for detection of suspected cases. CBS however, is an essential component of a measles elimination program as it provides data on the number of confirmed cases and characterizes the circulating wild-type virus[9].

In 2011, countries in the African region agreed to eliminate measles by 2020[10] by reducing the annual incidence of measles to <5 cases per 1,000,000 in the presence of an effective surveillance system [7]. However, as the incidence of measles decreases the PPV of any clinical case definition decreases [11].This study examined measles surveillance data between 2012 and 2015 in 112 districts of Uganda, to estimate the effectiveness and PPV of the measles surveillance system in order to improve efficiency and track progress to elimination.

## Methods

We analyzed eHMIS and measles case-based surveillance data between 2012 and 2015 to determine the effectiveness and PPV of the measles surveillance system.

A suspected case of measles was defined as any person with fever and generalized maculo-papular rash plus one of the following: cough, coryza or conjunctivitis; or any person in whom a clinician suspects measles. A **c**onfirmed measles case was a suspected case with positive IgM antibody or who was epidemiologically linked to a confirmed case in an outbreak.

We defined effectiveness of the measles case-based surveillance system as having ≥ 80% of suspected measles IDSR alerts with blood samples collected for laboratory confirmation [7]. The assessment of the effectiveness of a measles surveillance system requires that information reported through the eHMIS is validated by CBS to distinguish accurate from inaccurate alerts. To estimate the effectiveness of the measles case-based surveillance system, we compared two databases used for measles surveillance in Uganda [8]. First, the eHMIS which captures both the weekly measles surveillance and the monthly surveillance data in an Integrated Disease Surveillance and Response manner and the case-based surveillance with laboratory confirmation as the second data base, also considered as a reference standard for serological confirmation of measles. Effectiveness was computed by dividing the number of measles alerts with blood sample collected for laboratory confirmation during a particular year (numerator), by all the alerts of measles cases reported by eHMIS (denominator).

The PPV was defined as the proportion of measles case-patients identified by CBS who also had a positive measles serological result (IgM +) [12, 13]. It was computed by dividing the number of measles case-patients with serum positivity of measles IgM, by the total number of measles case-patients.

An IDSR alert was defined as ≥1 suspected measles case reported by a district through eHMIS in a week. An alert in the case-based surveillance system was defined as ≥ 1 suspected measles case with a blood sample collected for laboratory confirmation from a corresponding district during a particular week.

Measles surveillance data (2012–2015) were accessed from Uganda’s electronic Health Management Information System using DHIS2 software.

### Ethics

Approval to use the surveillance data was sought from Ministry of Health of Uganda which is responsible for utilization of health and health-related data/information in the health sector for the betterment of the population of Uganda. The office of the director general determined that this activity was not human subjects’ research and its primary intent was public health practice or a disease control activity (specifically epidemic disease control activity). To protect patient confidentiality, personal information were de-identified during extraction and data analysis. Therefore none of the authors had access to identifying information.

## Results

A total of 1,769 serum specimens were obtained from suspected measles case-patients, using the CBS in 2012. Of these, 61were excluded from further analysis because they had indeterminate serological evaluation. Of the remaining 1,708 suspected measles cases, 504 (28%) were measles specific IgM positive. Of the 1,190 suspected measles case-patients investigated by CBS in 2013, 21 (2%) had indeterminate serological evaluation. Of the remaining 1,169 suspected measles case-patients, 100 (8.6%) were measles specific IgM positive case-patients. Of the 1,652 suspected measles case-patients investigated by CBS in 2014, 20 (1.2%) had indeterminate serological evaluation and were not included in further analysis. Of the remaining 1,632 suspected measles case-patients, 144 (8.8%) were measles specific IgM cases-patients. Of the 2,363 suspected measles case-patients investigated in 2015, 78 (3.3%) had indeterminate serology results and were also excluded from further analysis. Of the remaining 2,285 suspected measles case-patients, 195 (8.5%) were measles IgM case-patients (Table 1).

**Table 1:** Mean age and sex distribution of measles cases using CBS for Uganda, 2012–2015.

Year	# IgM (+) measles cases	Male	Female	Median age (IQR)
		n %	n %	
2012*	504	244 (48)	260 (52)	2 (0.0–5.5)
2013	100	49 (49)	51 (51)	2 (1.0–9.0)
2014	144	74 (51)	70 (49)	1.0 (0.0–5.0)
2015	195	105 (54)	90 (46)	3.0 (0.0–7.0)
**2013–2015**	439	472 (50)	471 (50)	4.0 (1.0–6.0)

*Year with many measles outbreaks

In 2013,713 (63%) of the alerts reported in eHMIS had blood samples collected for laboratory confirmation. In 2014, the percentage of alerts with blood samples collected increased to 916 (84%) achieving the recommended WHO standard of ≥80%, this proportion however decreased in 2015 (Table 2). The PPV of the measles case-based surveillance between 2013 and 2015 was 8.6% (Table 3).

**Table 2:** Proportion of IDSR measles alerts with blood samples collected for confirmation in Uganda, 2012–2015.

Year	IDSR measles alerts (N)	Alerts with blood sample sent to lab (n)	% of alerts with blood sample collected
2012	190	850	N/A*
2013	1140	713	63
2014	1086	916	84
2015	1553	1083	70
2013–2015	3,969	3,562	72

*Year with inconsistences in reporting between weekly IDSR alerts and CBS alerts

**Table 3:** Positive predictive value of measles case-based surveillance for Uganda, 2012–2015.

Year	Number of suspected measles cases with blood samples collected for laboratory confirmation	# IgM (+) measles cases	PPV %
2012*	1769	504	30
2013	1190	100	8.6
2014	1652	144	8.8
2015	2363	195	8.5
**2013–2015**	**5205**	439	8.6

*Excluded from the calculation of the average PPV of measles CBS because of the high intensity of outbreaks during that year

## Discussion

Our review of measles surveillance data showed that the proportions of IDSR measles alerts with blood samples collected for laboratory confirmation (72%) through case-based measles surveillance were sub-optimal to detect measles outbreaks between 2013 and 2015. Secondly, the average PPV of the measles case-based surveillance system was within the recommended target of <10% by the World Health Organization African Region (WHO-AFRO) [7].

In 2012, all districts were transitioning from paper based reporting to electronic Health Management Information System [14]. During this period, most of the CBS alerts with blood specimen collected were different from the IDSR alerts of suspected measles cases reported through the eHMIS. The inconsistency in reporting between the weekly eHMIS and measles case-based surveillance could have been due to two reasons; first, most of the personnel in districts had not yet been trained in electronic reporting and therefore could have been unable to report using the system. Secondly, some districts had information technology challenges making it impossible to routinely report these cases. These data transmission challenges however, were resolved by Ministry of Health Resource Center and did not happen in subsequent years.

Surveillance systems that are functioning optimally should collect blood specimens from ≥ 80% of all suspected measles cases for laboratory confirmation if they are to detect outbreaks in time [15]. Having an effective surveillance system ensures that the information provided concerning low measles incidence is attributable to the absence of disease rather than to inadequate detection and reporting [16]. The inability of the system to perform at the required level during this period could have been due to several challenges inherent within surveillance systems [17]. Studies elsewhere have shown that some measles surveillance systems perform below average [18, 19]. However, it’s important to note that the implementation of case-based surveillance in Uganda has relied mainly on health workers who collect specimens from suspected measles cases that report to health centers with irregular visit to the community [20]. WHO-AFRO suggests that in order to overcome this limitation and get more accurate data about disease burden in the community, surveillance officers should regularly look for measles cases [19].

Other studies elsewhere have found similar results in measles surveillance systems[21, 22]. A study done in the World Health Organization European Region found that the majority of countries did not report routine use of laboratory confirmation for measles suspected cases [15].

The low PPV of the measles case-based surveillance system (8.6%) indicates that most of the suspected measles cases (19,620) that were reported between 2012 and 2015 were not true measles cases. This usually happens when the incidence of disease is rare. When the incidence increases the PPV also increases [23]. The low PPV also implies that serological confirmation of all measles cases in Uganda is important to ensure accurate diagnosis. As elimination of measles is approached it becomes critical to accurately identify each case of measles because it could be the only evidence of measles transmission in the area [12].

Among the suspected measles alerts that had blood samples collected for laboratory confirmation, the year 2012 registered the highest positive predictive value. During this period, several measles outbreaks were detected across the country before implementation of the measles mass campaign in November 2012. Following this campaign, the positive predictive value of the surveillance system reduced significantly.[24]. Although the PPV of CBS shows that measles incidence has significantly reduced, the median age of 4.0years, IQR (1.0–6.0), illustrates that measles transmission is still common in the under 6 year age-group. This may also necessitate measles mass campaigns and other interventions geared towards elimination to focus on this age group. A study done to illustrate the epidemiology of measles in the Region of America and Western Pacific Region showed that measles incidence may increase in older age groups in areas which have achieved elimination[25].

## Strength and limitations

Findings from this analysis should be interpreted with a grain of salt. A measles alert as used in this study does not put into consideration the number of cases reported during a particular week or the number of positive measles cases identified. Secondly, we lacked information external to the system to determine the true frequency of measles cases. The strength of this study was that we compared two national databases; the electronic Health Management Information System and the case-based surveillance system, which is the gold standard for measles diagnosis in Uganda.

## Conclusion

The effectiveness of measles case-based surveillance was sub-optimal, while the PPV showed that true measles cases have significantly reduced in Uganda. We recommended strengthening of case based surveillance to ensure that all suspected measles cases have blood samples collected for laboratory confirmation or line listed in case of confirmed outbreaks as an important part of measles control and elimination program in Uganda.
